# Supplementary material for: Scaling up of tsetse control to eliminate Gambian sleeping sickness in northern Uganda
Source: PLoS Negl Trop Dis. 2022 Jun 29;16(6):e0010222. doi: 10.1371/journal.pntd.0010222 (PMC9275725; doi:10.1371/journal.pntd.0010222)
Supplement: S1 Text — Supplementary Materials—Fig S1.1 Mean daily catch of tsetse in January-October 2014 in areas with (A) no targets, (B) annual deployment or (C) biannual deployment of targets. S1 Text: Supplementary Materials—Fig A. Schematic map of the arrangement of monitoring traps (red triangles) deployed in a transect along a drainage system (blue line) to quantify the impact of small-scale deployment of Tiny Targets. Dotted green line indicates the limits of a 7 x 7 km block within which targets were deployed in Phase 1. S1 Text: Supplementary Materials—Table A. Mean daily catch (95%CI) of tsetse from monitoring traps deployed along rivers transecting where Tiny Targets were deployed. S1 Text: Supplementary Materials—Table B. Mean daily catch (95%CI) of tsetse from monitoring traps deployed on Inve, Ayi and Oluffe rivers. S1 Text: Supplementary Materials—Table C. Mean daily catch (95%CI) of tsetse from monitoring traps deployed in areas with or without Tiny Targets. (DOCX) [file pntd.0010222.s001.docx]

**Supplementary Materials**

**1: Biannual** *vs***. Annual Deployment of Tiny Targets**

**Introduction**

In Uganda, Tiny Targets are normally deployed biannually. In 2014, we compared, for one year only, catches from monitoring traps in areas where targets were deployed once or twice a year or not at all.

**Materials and Methods**

Targets were deployed biannually (February and July) in the original (Phase 1) Inve, Aiivu and Kubala blocks (7 x 7 km) but annually (February only) elsewhere (Arua, Ayi, Ollufe) (14). The abundance of tsetse was assessed using Pyramidal traps, each operated for ~20 days/month with catches collected and counted at 24 h intervals. Traps were deployed at 34 sites in areas with a single deployment, 31 sites for areas with two deployments and at 15 sites along the Kochi river in Koboko district where no targets were deployed. For further details of trap locations and intervention areas, see main text and [1].

We hypothesized that reducing the number of deployments would lead to a statistically significant increase in the mean daily catch of tsetse. To compare catches from areas with biannual or annual deployment of targets, we produced a glmm with a negative binomial distribution, daily catch/trap as a response variable, day and site of capture as random effects, the number of deployments (0, 1, 2) as an explanatory factor, and month (1-10, numerical values for January-October) as a continuous explanatory variable. The statistical significance of explanatory variables was assessed using the Wald z-test.

**Results**

The results (Fig. 1) show that there was a significant effect of target deployment (Deviance=9.82, df=2, P=0.007; ANODEV), month (Deviance=1375.1, df=2, P<0.001) and a significant interaction between the two (Deviance=14.8, df=2, P<0.001). Catches in areas with targets were lowered significantly by the presence of targets deployed either annually (z=4.010, P<0.001) or biannually (z=4.356, P<0.001), but there was no significant difference in catch between annual and biannual deployments (z=0.3127, P=0.960). There was a significant (z=7.11, P<0.001) increase in catch with month (slope=0.0793, SE=0.0112) Thus, in areas with no targets, catches did not vary significantly with month (Fig. 1A; z=0.03, P=0.98). However, catches increased significantly with month for areas with annual (Fig. 1B; z=5.98, P<0.001) or biannual (Fig. 1C; z=5.16, P<0.001) deployment of targets. Pooling catches for areas with annual and biannual deployment of targets, mean daily catches increased at a rate of 0.1 (SE=0.02) tsetse/trap each month for the period January-October 2014.

**Conclusion**

We conclude that in the particular time and place of the present work, there was no material impact of deploying targets annually or biannually on the density or dynamics of the local tsetse population.

Figure S1.1 Mean daily catch of tsetse in January-October 2014 in areas with (A) no targets, (B) annual deployment or (C) biannual deployment of targets.

Fig A. Schematic map of the arrangement of monitoring traps (red triangles) deployed in a transect along a drainage system (blue line) to quantify the impact of small-scale deployment of Tiny Targets. Dotted green line indicates the limits of a 7 x 7 km block within which targets were deployed in Phase 1.


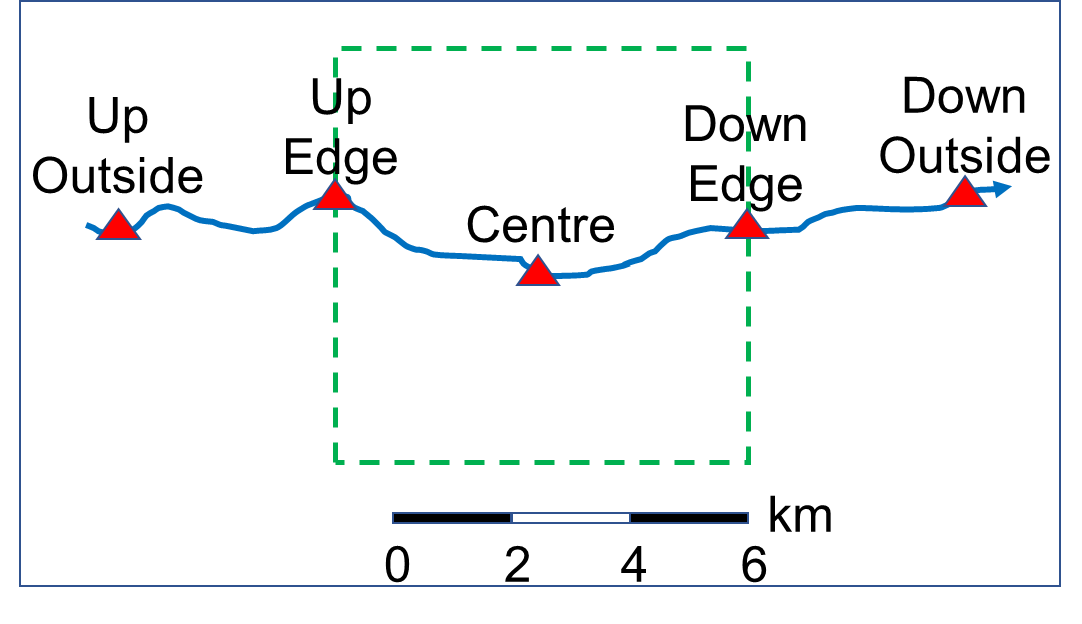


Table A. Mean daily catch (95%CI) of tsetse from monitoring traps deployed along rivers transecting where Tiny Targets were deployed.

| Location | Year | Mean | 95% CI | z value | P | Index |
| --- | --- | --- | --- | --- | --- | --- |
| Up, outside | 2011 | 2.12 | 0.306-14.740 |  |  | 1.000 |
|  | 2012 | 0.18 | 0.023-1.470 | -5.893 | <0.001 | **0.086** |
|  | 2013 | 0.03 | 0.004-0.261 | -9.856 | <0.001 | **0.015** |
|  | 2014 | 0.07 | 0.008-0.542 | -8.216 | <0.001 | **0.031** |
| Up, edge | 2011 | 12.70 | 4.484-35.965 |  |  | 1.000 |
|  | 2012 | 0.36 | 0.121-1.084 | -17.916 | <0.001 | **0.028** |
|  | 2013 | 0.10 | 0.032-0.321 | -21.200 | <0.001 | **0.008** |
|  | 2014 | 0.23 | 0.074-0.725 | -18.563 | <0.001 | **0.018** |
| Centre | 2011 | 4.04 | 2.385-6.845 |  |  | 1.000 |
|  | 2012 | 0.32 | 0.166-0.611 | -4.763 | <0.001 | **0.079** |
|  | 2013 | 0.08 | 0.039-0.172 | -8.376 | <0.001 | **0.020** |
|  | 2014 | 0.33 | 0.158-0.682 | -7.673 | <0.001 | **0.081** |
| Down, edge | 2011 | 8.43 | 4.689-15.141 |  |  | 1.000 |
|  | 2012 | 1.92 | 0.978-3.786 | -8.340 | <0.001 | **0.228** |
|  | 2013 | 0.61 | 0.304-1.223 | -14.157 | <0.001 | **0.072** |
|  | 2014 | 1.55 | 0.773-3.093 | -9.158 | <0.001 | **0.184** |
| Down, outside | 2011 | 11.60 | 7.434-18.087 |  |  | 1.000 |
|  | 2012 | 4.68 | 2.638-8.303 | -4.763 | <0.001 | **0.404** |
|  | 2013 | 2.03 | 1.094-3.758 | -8.376 | <0.001 | **0.175** |
|  | 2014 | 2.32 | 1.220-4.397 | -7.673 | <0.001 | **0.200** |
| No targets | 2011 | 2.37 | 1.556-3.622 |  |  | 1.000 |
|  | 2012 | 2.73 | 1.634-4.568 | 1.017 | 0.718 | **1.151** |
|  | 2013 | 2.47 | 1.498-4.071 | 0.272 | 0.992 | **1.040** |
|  | 2014 | 2.27 | 1.362-3.781 | -0.300 | 0.989 | **0.956** |

Catches for years 2012-2014, when targets were present, are expressed as a proportion (Index) of catches operated in 2011 (Sep-Oct only) before they were deployed. Probabilities (P) indicate that the Index is significantly different (z-statistic, Tukey contrasts) from unity. Catches for years 2012-2013 are the means for the period January and December and January-October for 2014. For monthly catches, see Fig. 2.

Table B. Mean daily catch (95%CI) of tsetse from monitoring traps deployed on Inve, Ayi and Oluffe rivers.

| River | Year | Months | Targets | Mean | 95% CI | z value | P | Index |
| --- | --- | --- | --- | --- | --- | --- | --- | --- |
| Ayi | 2011 | Sep-Oct | Absent | 12.97 | 52.055-3.233 |  |  | 1.000 |
|  | 2012 | Jan-Dec | Present | 0.29 | 1.271-0.067 | -12.021 | <0.001 | **0.022** |
|  | 2013 | Jan-Dec | Present | 0.02 | 0.100-0.004 | -17.419 | <0.001 | **0.002** |
|  | 2014 | Jan-Sep | Present | 0.02 | 0.092-0.003 | -16.961 | <0.001 | **0.001** |
| Inve | 2011 | Sep-Oct | Absent | 2.62 | 0.695-9.846 |  |  | 1.000 |
|  | 2012 | Jan-Dec | Present | 0.56 | 0.144-2.154 | -10.607 | <0.001 | **0.213** |
|  | 2013 | Jan-Dec | Present | 0.20 | 0.051-0.791 | -15.619 | <0.001 | **0.077** |
|  | 2014 | Jan-Sep | Present | 0.23 | 0.058-0.908 | -14.483 | <0.001 | **0.088** |
| Oluffe | 2012 | Jul-Oct | Absent | 15.86 | 7.575-33.191 |  |  | 1.000 |
|  | 2013 | Jan-Dec | Present | 1.31 | 0.557-3.080 | -10.589 | <0.001 | **0.083** |
|  | 2014 | Jan-Sep | Present | 0.50 | 0.197-1.249 | -13.657 | <0.001 | **0.031** |

For each River, catches for years when targets were present (2012-14) are expressed as a proportion (Index) of catches before they were deployed (2011). Probabilities (P) indicate that the Index is significantly different (z-statistic, Tukey contrasts) from unity. For monthly catches, see Fig. 4.

Table C. Mean daily catch (95%CI) of tsetse from monitoring traps deployed in areas with or without Tiny Targets.

| Block | Year | Mean | 95% CI | z value | P | Index |
| --- | --- | --- | --- | --- | --- | --- |
| Targets absent – Arua & Maracha | 2014 | 1.29 | 0.391-4.260 |  |  |  |
|  | 2015 | 1.65 | 0.442-6.121 | 0.870 | 0.644 | **1.437** |
|  | 2016 | 1.44 | 0.385-5.366 | 0.386 | 0.917 | **1.260** |
|  |  |  |  |  |  |  |
| Targets present - Arua | 2014 | 3.65 | 2.110-6.331 |  |  |  |
|  | 2015 | 0.45 | 0.221-0.916 | -8.935 | <0.001 | **0.145** |
|  | 2016 | 0.35 | 0.170-0.738 | -9.875 | <0.001 | **0.117** |
|  |  |  |  |  |  |  |
| Targets present - Maracha | 2014 | 2.76 | 1.758-4.335 |  |  |  |
|  | 2015 | 1.50 | 0.863-2.622 | -3.624 | 0.001 | **0.605** |
|  | 2016 | 1.17 | 0.665-2.070 | -5.014 | < 0.001 | **0.478** |
|  |  |  |  |  |  |  |
| Targets present - Koboko | 2014 | 3.33 | 2.188-5.068 |  |  |  |
|  | 2015 | 0.99 | 0.545-1.788 | -5.688 | <0.001 | **0.353** |
|  | 2016 | 0.93 | 0.503-1.701 | -5.942 | <0.001 | **0.336** |
|  |  |  |  |  |  |  |
| Targets present - Yumbe | 2014 | 3.35 | 1.460-7.707 |  |  |  |
|  | 2015 | 0.14 | 0.037-0.514 | -7.169 | <0.001 | **0.067** |
|  | 2016 | 0.28 | 0.081-0.966 | -5.733 | <0.001 | **0.125** |

Catches for years 2015-2016, when deployment of targets scaled up, are expressed as a proportion (Index) of catches operated in 2014 (Nov-Dec only) before scale-up. Probabilities (P) indicate that the Index is significantly different (z-statistic, Tukey contrasts) from unity. Catches for years 2015-2016 are the means for the period January December. For bi-monthly catches, see Fig. 5.

**Reference**

1. Tirados I, Esterhuizen J, Kovacic V, Mangwiro TN, Vale GA, Hastings I, et al. Tsetse Control and Gambian Sleeping Sickness; Implications for Control Strategy. PLoS Negl Trop Dis. 2015;9(8):e0003822. Epub 2015/08/13. doi: 10.1371/journal.pntd.0003822. PubMed PMID: 26267814; PubMed Central PMCID: PMCPMC4580652.
