# Supplementary material for: Scaling up of tsetse control to eliminate Gambian sleeping sickness in northern Uganda
Source: PLoS Negl Trop Dis. 2022 Jun 29;16(6):e0010222. doi: 10.1371/journal.pntd.0010222 (PMC9275725; doi:10.1371/journal.pntd.0010222)
Supplement: Attachment — Submitted filename: Hope et al Scaling up of tsetse control to eliminate Gambian sleeping sickness in northern Uganda MS FOR REVIEW PLoS NTD_jwh.pdf [file pntd.0010222.s002.pdf]

PLOS Neglected Tropical Diseases

Scaling up of tsetse control to eliminate Gambian sleeping sickness in northern Uganda

--Manuscript Draft--

**Manuscript Number:**	PNTD-D-22-00157
**Full Title:**	Scaling up of tsetse control to eliminate Gambian sleeping sickness in northern Uganda
**Short Title:**	Large-scale control of tsetse in Uganda
**Article Type:**	Research Article
**Keywords:**	Glossina; Trypanosoma; human African trypanosomiasis; vector control; Tsetse flies; Uganda; insect ecology, insect sampling, trap, insecticide-treated target

Abstract:

Background

Tsetse flies (Glossina) transmit Trypanosoma brucei gambiense which causes Gambian human African trypanosomiasis (gHAT) in Central and West Africa. Several countries use Tiny Targets, comprising insecticide-treated panels of material which attract and kill tsetse, as part of their national programmes to eliminate gHAT. We studied how the scale and arrangement of target deployment affected the efficacy of control.

Methodology and principal findings

Between 2012 and 2016, Tiny Targets were deployed biannually along the larger rivers of Arua, Maracha, Koboko and Yumbe districts in north west Uganda with the aim of reducing the abundance of tsetse by >70% and thereby interrupting transmission. The extent of these deployments increased from ~250 km^2 in 2012 to ~1600 km^2 in 2015. The impact of Tiny Targets on tsetse population was assessed by analysing catches of tsetse from a network of monitoring traps; ~~a~~ sub-~~sample~~ of captured tsetse were dissected to estimate their age and infection status. In addition, the condition of 780 targets (~195/district) was assessed for up to six months after deployment. In each district, mean daily catches of tsetse (G. fuscipes fuscipes) from monitoring traps declined by >80% to <1 tsetse/trap following the deployment of targets. There was no significant difference in the age structure of tsetse caught from traps within versus outside the intervention area. The median effective life of a Tiny Target was 61 (41.8-80.2, 95% CI) days.

Conclusions

Scaling-up of tsetse control reduced the population of tsetse by >80% across the intervention area. Even better control might be achievable by tackling invasion of flies from infested areas within and outside the current intervention area. This should involve deploying more targets, especially along smaller rivers, and extending the effective life of Tiny Targets.

**Additional Information:**	
**Question**	**Response**
**Financial Disclosure**<br><br>Enter a financial disclosure statement that describes the sources of funding for the work included in this submission. Review the submission guidelines for detailed requirements. View published research articles from *PLOS NTDs* for specific examples.	Funding for this research was provided by the Bill and Melinda Gates Foundation (www.gatesfoundation.org), with grants awarded to MJL (Grant ID#:1017770) and SJT (Grant ID#: OPP1104516), and the UK Biotechnology and Biological Sciences Research Council with grants awarded to SJT (BB/S01375X/1, BB/P005888/1, BB/L019035/1) The funders had no role in study design, data collection and analysis, decision to publish, or preparation of the manuscript.

Powered by Editorial Manager® and ProduXion Manager® from Aries Systems Corporation

This statement is required for submission and **will appear in the published article** if the submission is accepted. Please make sure it is accurate.<br><br>**Unfunded studies**<br><br>Enter: *The author(s) received no specific funding for this work.*<br><br>**Funded studies**<br><br>Enter a statement with the following details:<br>• Initials of the authors who received each award<br>• Grant numbers awarded to each author<br>• The full name of each funder<br>• URL of each funder website<br>• Did the sponsors or funders play any role in the study design, data collection and analysis, decision to publish, or preparation of the manuscript?<br>• **NO** - Include this sentence at the end of your statement: *The funders had no role in study design, data collection and analysis, decision to publish, or preparation of the manuscript.*<br>• **YES** - Specify the role(s) played.<br><br>* typeset	
**Competing Interests**<br><br>Use the instructions below to enter a competing interest statement for this submission. On behalf of all authors, disclose any competing interests that could be perceived to bias this work—acknowledging all financial support and any other relevant financial or non-financial competing interests.	The authors have declared that no competing interests exist.

Powered by Editorial Manager® and ProduXion Manager® from Aries Systems Corporation

This statement **will appear in the published article** if the submission is accepted. Please make sure it is accurate. View published research articles from *PLOS NTDs* for specific examples.<br><br>**NO authors have competing interests**<br><br>Enter: *The authors have declared that no competing interests exist.*<br><br>**Authors with competing interests**<br><br>Enter competing interest details beginning with this statement:<br><br>*I have read the journal's policy and the authors of this manuscript have the following competing interests: [insert competing interests here]*<br><br>* typeset<br><br>This statement is required for submission and **will appear in the published article** if the submission is accepted. Please make sure it is accurate and that any funding sources listed in your Funding Information later in the submission form are also declared in your Financial Disclosure statement.	
**Data Availability**<br><br>Authors are required to make all data	Yes - all data are fully available without restriction

underlying the findings described fully available, without restriction, and from the time of publication. PLOS allows rare exceptions to address legal and ethical concerns. See the PLOS Data Policy and FAQ for detailed information.<br><br>A Data Availability Statement describing where the data can be found is required at submission. Your answers to this question constitute the Data Availability Statement and **will be published in the article**, if accepted.<br><br>**Important:** Stating 'data available on request from the author' is not sufficient. If your data are only available upon request, select 'No' for the first question and explain your exceptional situation in the text box.<br><br>Do the authors confirm that all data underlying the findings described in their manuscript are fully available without restriction?	
**Describe where the data may be found in full sentences. If you are copying our sample text, replace any instances of XXX with the appropriate details.**<br><br>• If the data are **held or will be held in a public repository**, include URLs, accession numbers or DOIs. If this information will only be available after acceptance, indicate this by ticking the box below. For example: *All XXX files are available from the XXX database (accession number(s) XXX, XXX.*).<br>• If the data are all contained **within the manuscript and/or Supporting Information files**, enter the following: *All relevant data are within the*	All relevant data are within the manuscript and its Supporting Information files.

manuscript and its Supporting Information files.<br>• If neither of these applies but you are able to provide **details of access elsewhere**, with or without limitations, please do so. For example:<br><br>*Data cannot be shared publicly because of [XXX]. Data are available from the XXX Institutional Data Access / Ethics Committee (contact via XXX) for researchers who meet the criteria for access to confidential data.*<br><br>*The data underlying the results presented in the study are available from (include the name of the third party and contact information or URL).*<br>• This text is appropriate if the data are owned by a third party and authors do not have permission to share the data.<br><br>* typeset	
Additional data availability information:	Tick here if the URLs/accession numbers/DOIs will be available only after acceptance of the manuscript for publication so that we can ensure their inclusion before publication.

Manuscript Click here to access/download;Manuscript;Manuscript.pdf

Scaling up of tsetse control to eliminate Gambian sleeping sickness in northern Uganda

Short title: Large-scale control of tsetse in Uganda

Andrew Hope[1]*, Albert Mugenyi[2]*, Johan Esterhuizen[1], Inaki Tirados[1], Lucas Cunningham[1], Gala Garrod[1], Mike J Lehane[1], Joshua Longbottom[1], TN Clement Mangwiro[3], Mercy Opiyo[1,4], Michelle Stanton[1], Steve J Torr[1]*, Glyn A Vale[5], Charles Waiswa[2], Richard Selby[1]

[1]Liverpool School of Tropical Medicine, Liverpool, Merseyside, United Kingdom

[2]Coordinating Office for Control of Trypanosomiasis in Uganda, Kampala, Uganda

[3]Bindura University of Science Education, Bindura, Zimbabwe

[4]Barcelona Institute for Global Health, Hospital Clinic, Barcelona, Spain

[5]Southern African Centre for Epidemiological Modelling and Analysis, University of Stellenbosch, Stellenbosch, South Africa

*Corresponding Authors: Andrew Hope, Albert Mugenyi, Steve J Torr

E-mail: steve.torr@lstmed.ac.uk
ORCID iD: 0000-0001-9550-4030

Abstract

Background

Tsetse flies (*Glossina*) transmit *Trypanosoma brucei gambiense* which causes Gambian human African trypanosomiasis (gHAT) in Central and West Africa. Several countries use Tiny Targets, comprising insecticide-treated panels of material which attract and kill tsetse, as part of their national programmes to eliminate gHAT. We studied how the scale and arrangement of target deployment affected the efficacy of control.

Methodology and principal findings

Between 2012 and 2016, Tiny Targets were deployed biannually along the larger rivers of Arua, Maracha, Koboko and Yumbe districts in north west Uganda with the aim of reducing the abundance of tsetse by >70% and thereby interrupting transmission. The extent of these deployments increased from ~250 km^2 in 2012 to ~1600 km^2 in 2015. The impact of Tiny Targets on tsetse population was assessed by analysing catches of tsetse from a network of monitoring traps; a sub-sample of captured tsetse were dissected to estimate their age and infection status. In addition, the condition of 780 targets (~195/district) was assessed for up to six months after deployment. In each district, mean daily catches of tsetse (*G. fuscipes fuscipes*) from monitoring traps declined by >80% to <1 tsetse/trap following the deployment of targets. There was no significant difference in the age structure of tsetse caught from traps within versus outside the intervention area. The median effective life of a Tiny Target was 61 (41.8-80.2, 95% CI) days.

Conclusions

Scaling-up of tsetse control reduced the population of tsetse by >80% across the intervention area. Even better control might be achievable by tackling invasion of flies from infested areas within and outside the current intervention area. This should involve deploying more targets, especially along smaller rivers, and extending the effective life of Tiny Targets.

Author summary

Gambian human African trypanosomiasis (gHAT) is a neglected tropical disease caused by *Trypanosoma brucei gambiense* transmitted by tsetse flies (*Glossina*). Uganda's strategy to eliminate gHAT includes the deployment of Tiny Targets, comprising insecticide-treated panels of cloth which attract and kill tsetse. Our data from a network of monitoring traps assessed how increasing the intervention area from ~250 km^2 to ~1600 km^2 affected the degree of control. Inspection of deployed targets indicated their effective lifespan. Targets reduced tsetse abundance by >80% beside the rivers where they were deployed but had no clear effect on adjacent tributaries where targets were absent. As the intervention area increased, so did the extent of the area controlled. We did not deploy targets along the smaller rivers and, as expected, the tsetse population was not eliminated. Our findings suggest that the population was sustained at low levels by invasion of tsetse from untreated parts of the drainage system. The average effective life of targets was ~60 days as against the ~180 days for targets deployed in Kenya. This discrepancy is attributable, in part, to the Uganda targets being removed by seasonal floods. While the level of control achieved is already more than sufficient to interrupt transmission of gHAT, even better control would be achieved by increasing the coverage of the drainage system.

Introduction

Sleeping sickness (human African trypanosomiasis, HAT) is caused by sub-species of *Trypanosoma brucei* spread by tsetse flies (*Glossina*). In the 20th Century there were three major epidemics of HAT, the most recent being in the late 1990s when up to >35000 cases were reported annually[1]. WHO declared the aim of eliminating HAT as a public health problem by 2020 and the complete interruption of transmission by 2030. Over the past 20 years, the number of cases reported globally has declined by >95%, with <2000 cases reported annually since 2017. WHO has formally validated the successful elimination of HAT as a public health problem in Côte d'Ivoire and Togo, where cases of HAT have been <1 case per 10000 inhabitants in all districts during a five-year period.

Most (>95%) cases of HAT are caused by *T. b. gambiense*, giving rise to gambiense HAT (gHAT), transmitted by riverine species of tsetse. Sub-species of *Glossina fuscipes* are the major vectors across a large swathe of Central Africa, extending across South Sudan, Uganda, the Democratic Republic of Congo (DRC), Central African Republic (CAR) and Chad [2]. Together, these countries contributed >90% (27296/32275) of all cases of gHAT reported globally in the period 2011-2020[1].

The current low incidence of the disease has been achieved largely through the mass screening and treatment of the human population. However, modelling and empirical evidence suggests that vector control can make an important contribution to achieving the elimination goals [3, 4]. The development of effective and affordable methods of tsetse control makes such an integrated approach feasible. In places where cattle are present in adequate densities (>10 cattle/km^2), treatment of cattle with pyrethroids to control tsetse can be highly cost-effective [5, 6]. In areas with lower densities of cattle, the deployment of insecticide-treated targets which attract and kill tsetse is recommended [7, 8].
An important consideration in the use of insecticide-treated cattle and targets is their spatial distribution. Tsetse can displace between ~200 and ~1000 m/day [9] modulated by local topography [10]. The mobility of tsetse has two important implications for vector control. On one hand, small scale operations, covering say a single village within 4 km^2 will be ineffective because tsetse from neighbouring uncontrolled areas re-invade rapidly, so that there is no perceptible change in the numbers of tsetse [11]. On the other hand, the mobility of tsetse allows the sparse deployment of baits because a fly's movement will bring it to the vicinity of a bait.

Savanna species of tsetse, which include important vectors of *T. b. rhodesiense*, are highly mobile, displacing ~500-1000 m/day [11]. They are distributed widely across savanna woodland and are highly responsive to artificial odour attractants [12]. To control these species, odour-baited targets are deployed evenly at low densities (e.g., 4 targets/km^2), but the intervention must be applied over large areas of at least 500 km^2 to reduce invasion problems [13] [9].

The important riverine vectors of *T. b. gambiense* are far less responsive to artificial odour attractants [14, 15] such that targets for them are used generally without odours. This requires target densities to be increased several-fold in compensation. Against these problems there are four important benefits in dealing with riverine tsetse. First, these species are relatively immobile, displacing at ~300 m/day [16], so that it becomes feasible to consider control in relatively small areas, i.e., <50km^2 [17]. Second, the riverine flies are restricted to the vegetated fringes of rivers and lakes, so that the treated areas can be decreased yet further. Third, it is not necessary to reduce the density of riverine tsetse to

zero in order to break the transmission cycle of gHAT; reducing the population by ~70% is sufficient for this [17]. Finally, whereas the targets for savanna tsetse should be least 1m^2, the riverine flies can be tackled using targets only ~0.1m^2, i.e., the so-called Tiny Targets [18]. Such targets have been particularly effective against riverine species of tsetse in the gHAT foci of Uganda [17], Chad [4], Guinea [19], DRC [20] and Côte d'Ivoire [21]. Epidemiological analyses have also shown that deployment of Tiny Targets reduced the incidence of gHAT in Guinea [22], Chad [4] and Uganda [23].

Methods used to control trypanosomes and tsetse each have their own strengths and weaknesses. For control of vectors, one of the biggest problems is arguably the re-invasion of tsetse from uncontrolled areas nearby. It is usually too costly and impractical to avoid this problem by eliminating tsetse within the invasion sources because such sources are commonly very extensive in comparison to the foci where gHAT occurs. A better understanding of how the level of suppression is affected by the degree to which control operations cover the drainage systems would provide a rational basis for optimising strategies for controlling tsetse. The general principles governing the relationships between the scale of control operations, density of targets and mobility of tsetse are understood but we have little empirical evidence to guide the design of control campaigns applicable to specific ecological and epidemiological settings.

Uganda was one of the first countries to use Tiny Targets successfully in an area affected by gHAT [17]. Between 2011 and 2012 (Phase 1), targets were deployed across five separate 7 × 7 km blocks of Arua and Maracha districts in north west Uganda. In early 2013 (Phase 2), the control operation expanded to produce a single operational area of 500 km^2. The operation resulted in a >90% reduction in numbers of tsetse caught by traps in the areas where targets were deployed. From January 2015 onwards (Phase 3), tsetse control in north west Uganda was expanded further to form an operational area of ~2500 km^2 covering most of the HAT-affected areas of Arua, Maracha, Koboko Yumbe and Moyo districts [24]. The efficacy of these control operations has been monitored consistently using a network of tsetse traps.

In previous papers, we have reported the impact of these control operations on tsetse populations [17], their economic cost [25], the perceptions of local people to baits [26], and the impact on gHAT cases [23]. Here we use data from entomological monitoring to assess how the various scales (50-1600 km^2) of control operations conducted across a contiguous area covering parts of Arua, Maracha, Koboko and Yumbe districts, impacted on the distribution and abundance of tsetse populations within and outside the intervention areas. In particular, we address the following three questions:

1. What was the impact of small-scale (50 km^2) interventions on the abundance of tsetse in neighbouring untreated areas?

2. Did expansion of tsetse control operations produce higher levels of suppression within the control area and alter the population age structure of tsetse?

3. What is the operational lifetime of Tiny Targets when deployed in a large-scale tsetse control programme?

Materials and Methods

Ethics statement

The research did not involve any human participants, domestic livestock or wild vertebrate animals. The data were produced under the auspecies of the Uganda National Council of Science and Technology ethics board ("Targeting tsetse: use of targets to eliminate African sleeping sickness" Ref. Number HS 939) and a Memorandum of Understanding between the Uganda Trypanosomiasis Control Council (UTCC) and the Liverpool School of Tropical Medicine ("Tsetse control programme using insecticide-treated Tiny Targets in the West Nile HAT foci of Uganda (Moyo, Koboko, Arua, Maracha and Yumbe", ADM.7/166/01).

Research questions

1. Impact of small-scale interventions on neighbouring untreated areas. We hypothesized that tsetse are confined to rivers and that the flies move mainly along rather than between rivers. Accordingly, we expected that small-scale interventions applied to a particular river system would impact ~~on~~ tsetse populations on that river but not adjacent ones. To test this hypothesis, we compared the catches from traps deployed in a transect across the original small (7 × 7 km) intervention blocks to assess whether the overall impact varied according to local hydrography. We also monitored the abundance of tsetse in rivers adjacent to those where Tiny Targets were deployed, to assess whether the impact of targets was extended beyond the deployment rivers.

2. Impact of scale-up on levels of suppression and population age structure. As the extent of control operations expanded, we expected that improved levels of control might be achieved because the proportion of the area subject to re-invasion would be reduced. To elucidate this, we compared the catches from a network of traps deployed across the intervention area. We hypothesized that traps near the edge of the operational area would have catches greater than those at the centre if the operational area were invaded at a significant rate from outside, as against the redistribution of flies between the treated and untreated rivers within the operational area. In addition to reducing the catch of tsetse, we also assessed whether the age structure of the tsetse population was altered; since targets increase the local death rate of tsetse we predicted that the control measures would reduce not only the numbers of tsetse but also the mean age of the population if invasion were not important. This prediction is in accord with the theoretical [27] and practical [28] aspects of target performance.

3. Performance of Tiny Targets in large-scale operational settings. As the intervention area increased, the operation transitioned from being an externally managed research project to being managed by the Co-ordinating Office for Control of Trypanosomiasis in Uganda (COCTU) and implemented by District Entomologists rather than researchers and technicians. Monitoring the deployment of targets and their impact on tsetse populations allowed us to assess whether scaling up affected the overall quality of target deployment and the impact on tsetse populations.

Study area

All field studies were carried out in Arua, Maracha, Koboko and Yumbe districts in north west Uganda where gHAT has been a long-standing health problem -- with 2928 cases being reported in the period 2001-2010 [29]. These districts are within the West Nile region, bordering South Sudan and the Democratic Republic of the Congo, and mostly comprise small-scale arable farms producing cassava, millet, peanuts and tobacco, with relatively few

cattle and pigs [30]. There are two perennial rivers (Enyau, Kochi) and numerous smaller (<3 m wide) tributaries and streams, some of which cease flowing during the dry season (January-March). Narrow (~2-5 m) bands of natural vegetation (e.g., *Cynometra alexandri*, *Entada abyssinica*, *Acacia seyal*) along the banks of the rivers and streams provide habitat for tsetse. Wild mammalian hosts are rare in the area but Nile Monitor lizards are present and form part of the diet of tsetse, along with humans, cattle and pigs [31].

Deployment of Tiny Targets

Tiny Targets [18, 32] have been used in north west Uganda since 2011 [17] with the scale of control being increased in three phases (Fig. 1). For each phase, Tiny Targets were deployed along the rivers and larger streams at 100 m intervals on both banks giving a density of 20 targets/km along treated rivers. In general, targets were deployed at six month intervals because previous work had shown that the performance of the insecticide declined markedly after six months [17]. Between November 2011 and November 2012, Phase 1, targets were deployed in five separate blocks, each block being a 7 × 7 km square, producing a nominal total coverage of ~250 km^2 (Fig. 1A). From December 2012, Phase 2, targets were deployed over a larger area of, nominally, 500 km^2 which consisted of a convex hull encompassing the initial five blocks (Fig.1 B). In 2014, the first deployment was carried out successfully in January-March but the second deployment, conducted in July 2014, was incomplete with targets being deployed in the original (Phase 1) Inve, Aiivu and Kubala blocks but not elsewhere. Finally, between January 2015 and December 2016, Phase 3, the extent of operations expanded to a nominal area of 1636 km^2 (Fig. 1C). In Phases 1, 2 and 3, the total number of Tiny Targets deployed every six months were ~1300, ~2900 and ~14100 targets, producing overall densities of 5, 6 and 9 targets/km^2, respectively (Fig1 D-F).

FIG. 1 HERE

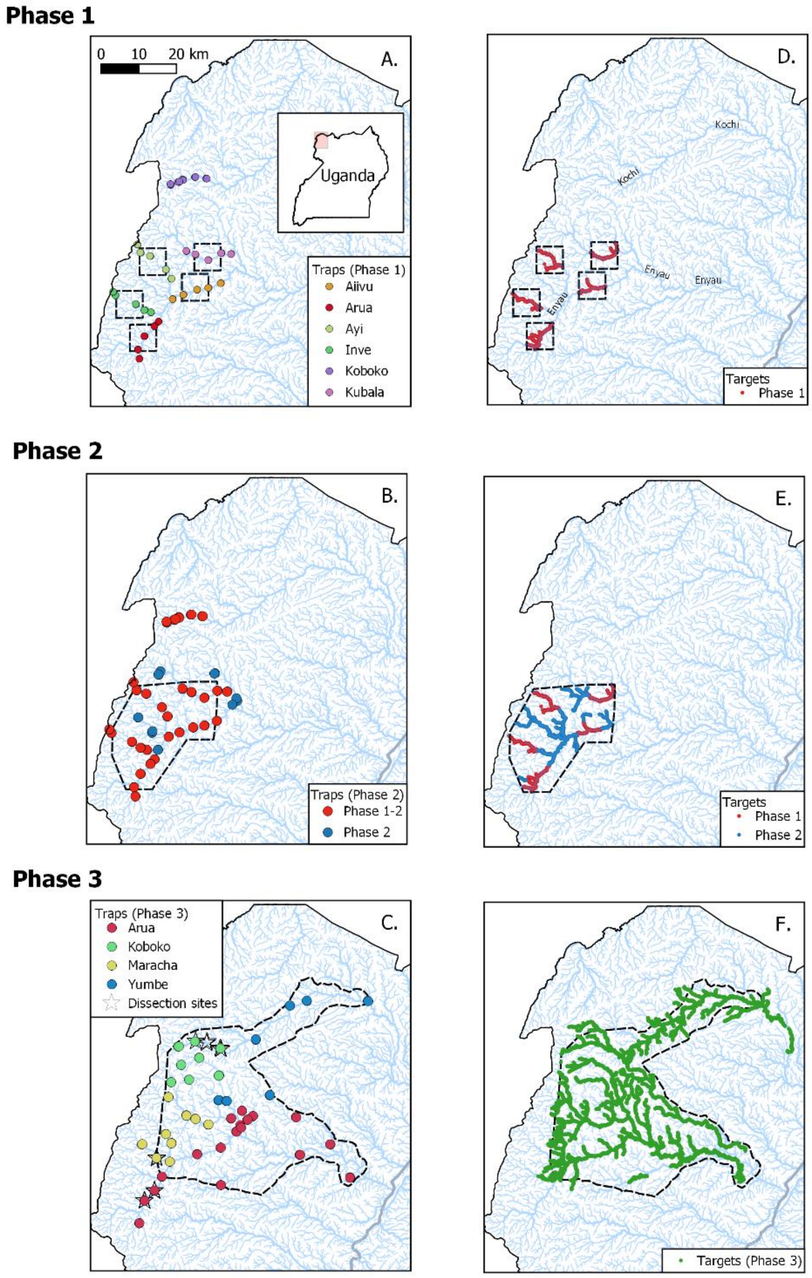

Fig. 1. Extent of planned tsetse control operations (black polygons with dashed lines) and locations of monitoring traps and Tiny Targets deployed between 2011 and 2016.
(A) Phase 1 (November 2011- November 2012): five square blocks, each 7 × 7 km. (B) Phase 2 (December 2012 – December 2014): single polygon of 500 km^2). (C) Phase 3 (January 2015 – December 2016): single polygon of 1636 km^2. For C only, monitoring traps with stars indicate sites where tsetse were collected for dissection. D-F show, in (D) red, (E) red & blue or (F) green where targets were deployed in Phases 1-3; the untreated rivers and streams are shown in pale blue.

Entomological monitoring

Tsetse populations were monitored using pyramidal traps [33]. The numbers of monitoring sites expanded with the extent of the control operation (Fig. 1). For Phase 1, pairs of traps, 100 m apart from each other, were deployed at the centre, edge and ~2 km outside the 7 × 7 km blocks, so providing a total of 50 monitoring traps in or close to areas where targets were deployed. For Phase 2, a further 14 traps were added to the existing ones to provide 64 traps across the 500 km^2 intervention area. For Phases 1 and 2, 14 traps were also deployed along the Kochi river in Koboko district (northern most traps in Fig 1, A & B) where no targets were deployed. These traps provided a measure of the underlying seasonal variation in the abundance of tsetse in the absence of Tiny Targets. Finally, in Phase 3, a network of 82 traps was used to monitor tsetse across 1636 km^2. For Phase 3, the expansion of tsetse control operations included the Kochi river but traps were routinely operated along sections of the Oluffe river in Maracha district, and the Enyau river in Arua district where targets were not deployed. These sites were in areas that had been originally subject to control during Phase 2 and the catches provided a measure of the response of a tsetse population to the cessation of control.

For all phases, daily catches from traps were collected, counted and identified to species and sex, for 5-20 days per month. Tsetse collected from a sub-sample of 15 traps (Fig. 1C, stars) were regularly dissected to provide estimates of infection status and age in areas with or without Tiny Targets.

Monitoring Tiny Targets

The locations of all Tiny Targets were recorded with a global positioning system (GPS, Garmin eTrex) as the targets were deployed and these data were mapped using QGIS, an open-source geographical information system (GIS). In 2015, we randomly selected ten 1-km sections of rivers in each district where targets had been deployed; sections were excluded if they were (i) >1 km (~10 min walk) from a vehicle access point, (ii) included a village or (iii) required walking for >30 mins to cover the length of the river. Each section of river had ~20 targets. GPS records of each target's location and its date of deployment were used to estimate how long each target had been in the field. The condition of each target located was recorded, i.e., whether it was present, upright and undamaged.

Statistical analyses

Catches from traps were analysed using the open-source statistical software R [34]. We used the 'glmmADMB' package to fit generalized linear mixed models (glmm) to catch data using a negative binomial data distribution and a log link function. To analyse the overall trends following the deployment of targets, the site and day of capture were specified as random effects and months as a fixed effect. For analyses where we assessed the statistical significance of differences in proportions (e.g., proportion of targets functioning correctly), we fitted data to a general linear model (glm) with a binomial error distribution and a logit link function. The statistical significance of differences in catches or proportions were assessed using the 'glht' function from the 'multcomp' package. We report predicted means and their 95% Confidence Intervals based on model parameter estimates.

Results

Impact of small-scale interventions on neighbouring untreated areas

Re-invasion of small intervention areas. Following the deployment of Tiny Targets in relatively small (7 × 7 km) areas, the abundance of tsetse declined within and outside those areas (Fig. 2). The decline in catch varied according to the location of the traps relative to the intervention area. Traps on the upstream side of the block or centre produced the lowest catches, with mean daily catches declining to <0.1 tsetse/trap, whereas those on the downstream sides produced minimum daily catches of >0.1 tsetse/trap (Fig. 2D, downstream edge) or >1 tsetse/trap (Fig. 2E, downstream outside). The reduction in catches 6-12 months after targets were first deployed were 86% and 77% for the traps outside-upstream and outside-downstream respectively, compared to 96% at the centre. These general patterns persisted when targets were deployed more widely in Phase 2 (months 15-38) with 97% control being achieved upstream, 90% downstream and 95% at the centre. The improved level of control in Phase 2 is because some of the monitoring traps that were outside the Phase 1 blocks were inside the Phase 2 intervention area. While catches declined by >90%, we continued to catch tsetse, albeit in low numbers, at all sites. Towards the end of 2014 (months 30-38), catches at all sites increased slightly. This may be related to an incomplete deployment of targets in mid-2014 but numbers of tsetse did not recover to their pre-intervention levels.

FIG 2 here

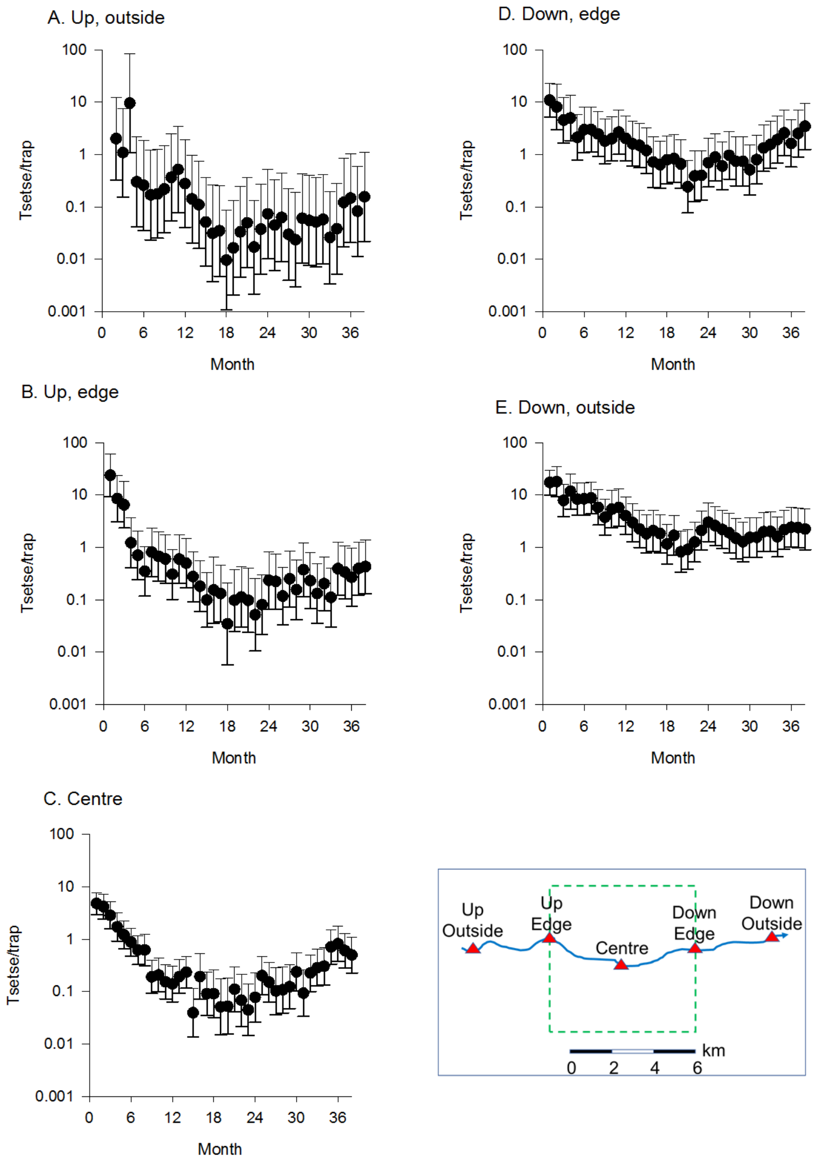

Fig. 2 Mean daily catch of tsetse from traps transecting Phase 1 intervention blocks.

For each intervention block, pairs of traps were located at sites (A) upstream and outside, (B) on the upstream edge, (C) at the centre, (D) downstream edge and (E) downstream and outside. Inset shows locations of traps, river and the intervention block schematically.

Impact of Tiny Targets on neighbouring catchments. Following the deployment of targets on the Ayi, mean daily catches of tsetse declined by 99%, from 16 tsetse/trap over the period September-October 2011(months 1-2) to 0.2 tsetse/trap for the period May 2012 - October 2014 (months 9-14) (Fig. 3A). On the Inve, mean daily catches declined by 82% from 3 tsetse/trap before targets were deployed to 0.6 tsetse/trap for the period May 2012 – October 2014. While catches on the Ayi and Inve were both <1 tsetse/day during the latter half of 2012, those on the adjacent and untreated Oluffe were 40 times greater, averaging 15 tsetse/day (Fig. 3C). The higher catches on the Oluffe suggest that tsetse populations were not affected by targets deployed on the adjacent Ayi and Inve rivers.

Targets were deployed on the Oluffe from November 2012 onwards and thereafter the catches declined (Fig. 3C) to ~1 tsetse/day from July 2013 onward, similar to catches on the Ayi and Inve. Overall, the results suggest that the effects of targets are restricted to the rivers where they are deployed and have little effect on tsetse populations in adjacent tributaries 3-5 km away.

FIG 3 here

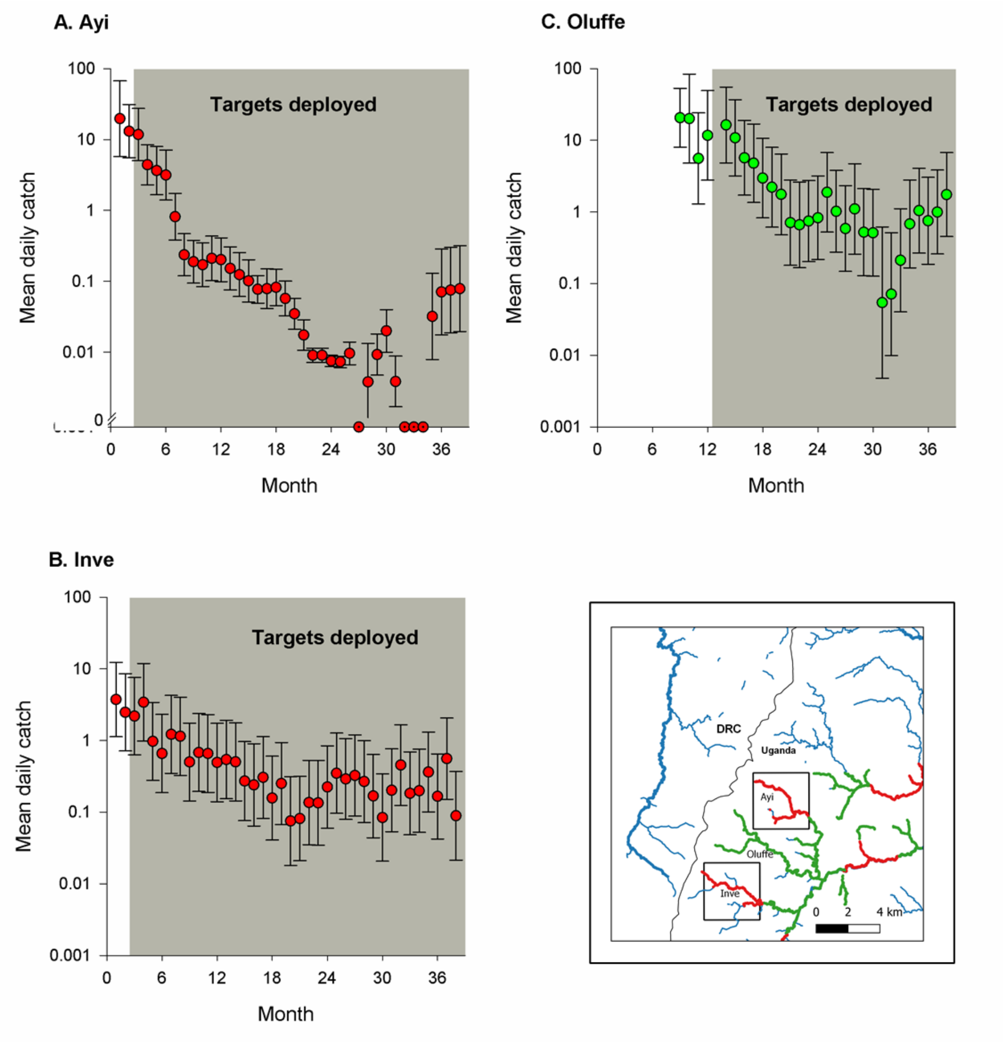

Fig. 3. Catches of tsetse on the (A) Ayi, (B) Inve and (C) Oluffe rivers.

Grey areas denote when targets were present. Inset shows a map of the Ayi, Inve and Oluffe rivers and the distribution of Tiny Targets deployed during Phase 1 (red sections) and Phase 2 (red and green) of the intervention.

Impact of large-scale tsetse control operations

From January 2015 onwards, the area over which Tiny Targets were deployed expanded from ~500 km^2 (Fig. 1B, E) to ~1600 km^2 (Fig. 1C, F). Logistical constraints meant that we were unable to operate traps at a standard frequency in each district every month. Moreover, national holidays associated with Christmas and New Year reduced the number of sampling days available in December and January. We therefore used two-month periods (i.e., November & December, January & February, etc.) to estimate temporal changes in the abundance of tsetse following the deployment of Tiny Targets.

FIG. 4 here

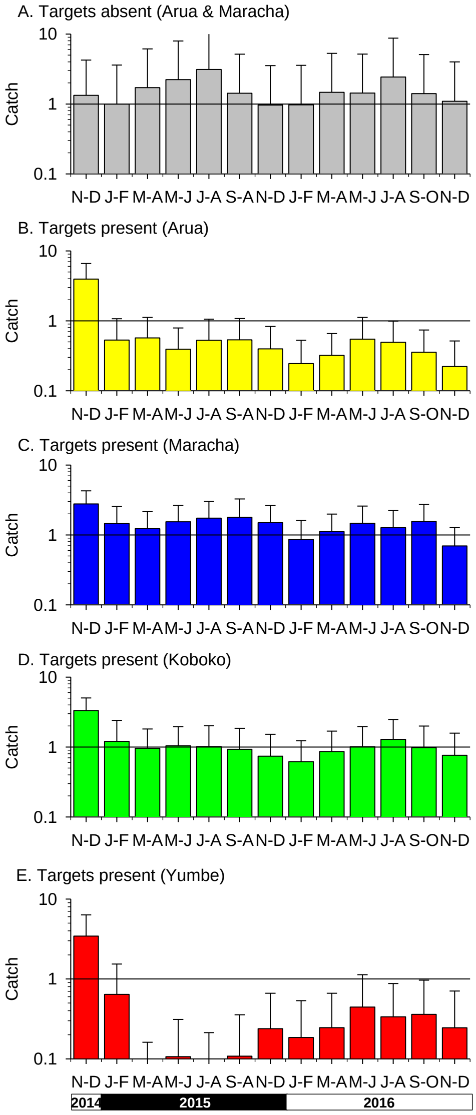

Fig. 4. Mean daily catches of tsetse from monitoring traps in areas with or without Tiny Targets from November 2014 – December 2016.

The results from monitoring traps (Fig. 4) show that on rivers in Arua and Maracha districts, at sites 4-14 km upstream of rivers where Tiny Targets were deployed, the mean catch of tsetse remained at >1 tsetse/day throughout the monitoring periods (November 2014-

December 2016) (Fig. 5A). On rivers where Tiny Targets were deployed, mean daily catches declined to <1 tsetse/trap (Arua, Yumbe) or ~1 tsetse/trap in Koboko, but in Maracha there was no marked and sustained decline. Expressing catches in the period July 2015 – November 2016 (i.e., >6 months after targets were deployed in Phase 3) as a percentage of catches in November-December 2014 (i.e., before targets were deployed), catches declined by 92% in Yumbe, 90% in Arua, 73% in Koboko and 52% in Maracha. For traps deployed in areas without targets, catches increased by 19%. Although catches declined in all districts where targets were deployed, nowhere did the catches become zero for extended periods. For instance, four traps located in the original Kubala block of Phase 1, which itself was near the centre of the Phase 3 intervention area (see Fig. 1A and C), caught a total of 78 tsetse in 136 trap-days in 2016 (mean 0.57 flies per trap day).

Analyses of the ovarian age of tsetse captured in areas with or without targets present showed that there was no significant difference in the age structure even in 2016 when targets had been deployed for >12 months (Fig.5). These results show that while the intervention reduced the abundance of tsetse, it did not alter age structure. Similarly, the infection rates of tsetse in the two areas were not significantly different (Table 1).

FIG. 5 here

Table 1. Percentage of tsetse captured in 2015-2016 with trypanosomes observed in salivary glands, midgut or mouthparts from areas with targets present or absent.

Targets	*n*	Salivary glands	Midgut	Mouthparts
Present	2619	0.04%	0.46%	0.92%
Absent	4790	0.00%	0.08%	1.52%

. cii proportions 4790 4
Binomial Exact --
Variable Obs Proportion Std. Err. [95% Conf. Interval]

4,790 .0008351 .0004174 .0002276 .0021367
. cii proportions 2619 12
Binomial Exact --
Variable Obs Proportion Std. Err. [95% Conf. Interval]

2,619 .0045819 .0013196 .0023697 .00799

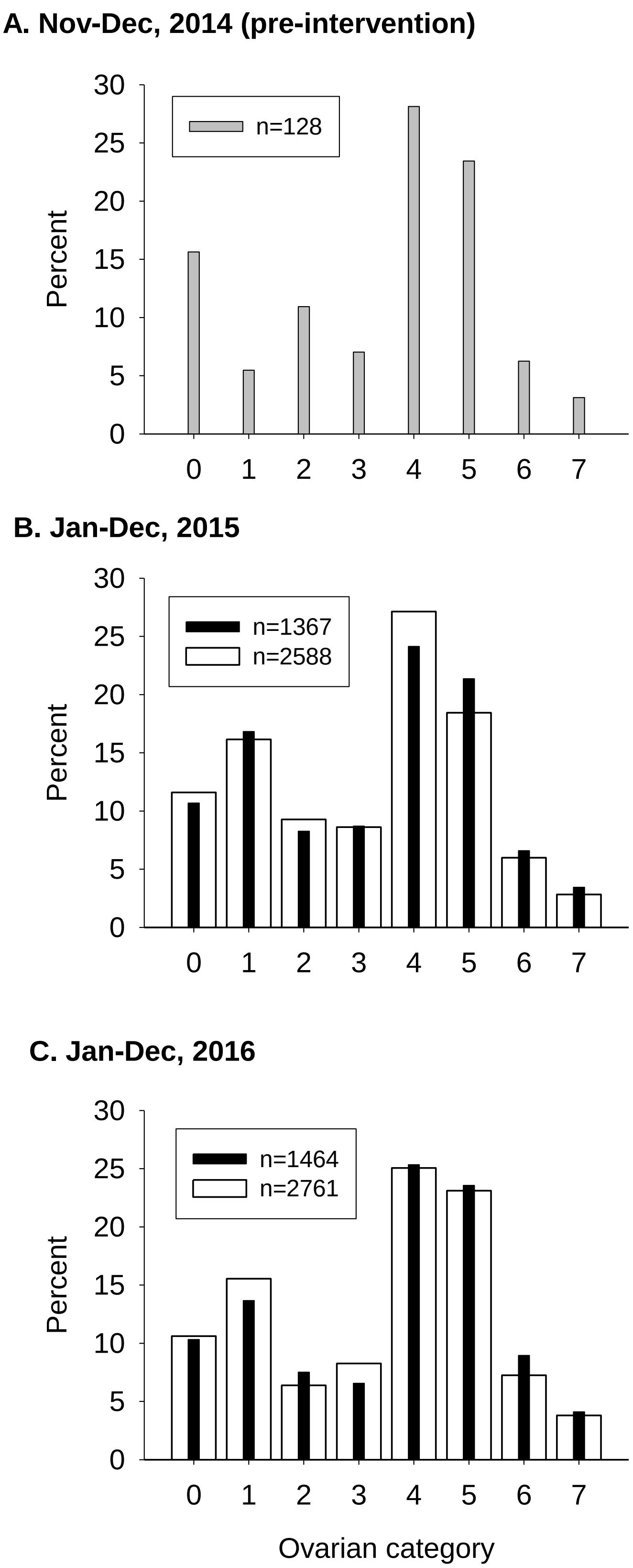

Fig. 5. Ovarian age structure of tsetse (A) before Tiny Targets with deployed and (B,C) subsequently in areas with (solid bars) or without (open bars) Tiny Targets.

Performance of Tiny Targets in large-scale operational settings

We inspected the condition of 780 targets across four districts and found that there was a significant effect of days since deployment and proportion of targets functioning ($F=8.5$, $P<0.001$); 61% were functioning at four weeks after deployment declining to <20% by 22 weeks (Fig. 6A). There was also significant variation between districts ($F= 6.5$, $P<0.001$,) with Maracha and Arua having a significantly lower proportion of functioning targets than either Koboko or Yumbe (Fig. 6B). There was no significant interaction between the effects of target age and district ($F=1.6$, n.s.). Overall, 42% (322/775) of targets inspected were functioning, 11% (85) were damaged, 21% (159) had fallen over and 27% (209) were missing.

Fig. 6 here

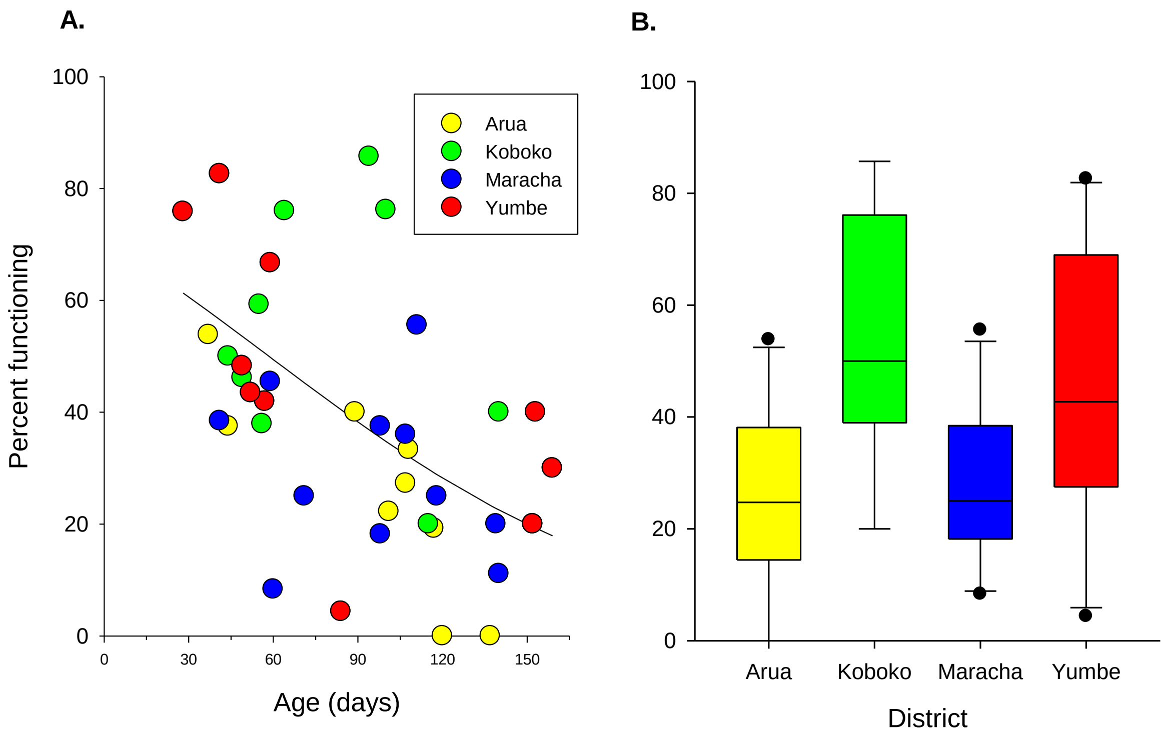

Fig. 6. (A) Scatterplot of percentage of targets functioning against age (days since deployment) and (B) boxplot of percentage functioning for each district.

Discussion

Scaling-up tsetse control: impact on tsetse abundance

The development of target deployment from a small-scale trial, managed and implemented by researchers, to a large-scale operation managed by a national programme and implemented by District Entomologists was successful; targets were deployed regularly at six month intervals covering most of the intended 1600 km^2. In accord with previous studies of the impact of Tiny Targets on *G. f. fuscipes* in Uganda [17], we show that the deployment of targets in new districts (Yumbe, Arua) led to a >80% decline in the catches of tsetse from monitoring traps. Similarly, catches from monitoring traps deployed in Koboko also declined by >70% but the decline was less marked in Maracha (52%). Prior to the deployment of targets, the mean daily catch from traps deployed in Maracha was 2.8 tsetse/day and catches in the following months ranged between 0.7 and 1.8 tsetse/day, representing declines of between 75% and 46% respectively (Fig. 4). The smaller effect in Maracha may be due, in part, to the impact of earlier deployments of Tiny Targets during Phases 1 and 2, i.e., operations conducted in 2011-2014. These covered large parts of Maracha district (see

Fig. 1) and previous analyses of this intervention [17] showed, for instance, that the mean daily catches from monitoring traps operated in the Kubala block of Maracha district (Fig. 1A) caught 12.4 tsetse/trap in September 2011, before targets were deployed. Catches subsequently declined to 0.3 tsetse/trap by June 2012 [17]. In the present study the catches from traps within the original Kubala block caught a total of 78 tsetse over 136 trap-days (arithmetic mean=0.6 tsetse/trap/day) in 2016. Hence comparing the catches in 2015-2016, when targets were present, against catches in late 2011, suggests that there has been a >90% decline in the abundance of tsetse since Tiny Targets were first deployed.

Impact of Tiny Targets across the drainage system

In addition to confirming that catches of tsetse declined along rivers where Tiny Targets were deployed, we also found that catches declined in contiguous parts of the same river where targets were not deployed (Fig. 2), but there was much less impact on catches from monitoring traps on an adjacent tributary (Fig 3). Previous studies have also reported the impact of targets on tsetse populations outside an intervention area for both savanna [13] and riverine [35] species. The finding that the impact of Tiny Targets is mostly along rivers rather than between them accords with studies of marked tsetse which showed that few riverine tsetse moved between rivers [36]. In our study, the impact of Tiny Targets was greater at upstream parts of the river system. These patterns are probably related to the natural movement and abundance of tsetse. Analyses of factors affecting the abundance of riverine tsetse have shown that in this part of Uganda catches of tsetse are strongly correlated with the overall density of rivers and associated vegetation [35]. In upper parts of the river network, streams are smaller and seasonal, and associated vegetation is reduced. Consequently, tsetse abundance is lower and fewer tsetse can re-invade an intervention area.

The evidence that deployment of targets on the Ayi and Inve rivers did not have an impact on tsetse populations on the Oluffe assumes that the densities on these three tributaries of the Enyau were broadly similar before targets were deployed. Evidence to support this assumption is provided by the results of a tsetse survey conducted in this area in October-November 2010, before targets were deployed, which showed that catches from traps deployed on these rivers were broadly similar [35]. This finding suggests that tsetse populations may persist if targets are not deployed on all rivers.

The present results also provide first information on the impact of removing targets on a tsetse population. The traps in areas without targets in Phase 3 were located in parts of Arua and Maracha where targets were present in Phases 1-2. The mean daily catches in these areas, after cessation of control, ranged between 1.0 and 3.1 tsetse/trap (Fig. 4A). The traps in Maracha were deployed on Oluffe and caught 5.6-20.6 tsetse/trap before targets were deployed (Fig. 3C) and those in Arua caught 9.0 tsetse/trap [17]. These results suggest that withdrawal of targets from western/upstream parts of Arua and Maracha in January 2015 did not result in tsetse recovering rapidly to their pre-intervention levels during the 24 month sampling period (January 2015 – December 2016).

Models of tsetse populations re-bounding following complete cessation of control suggest that the population will recover to ~50% of its pre-intervention abundance after 24 months [17]. We suggest that the tsetse population did not recover as rapidly as models predict because the large Phase 3 intervention in the downstream areas reduced the numbers of tsetse, preventing re-population of the upstream sections.

Impact of Tiny Targets on age structure of tsetse populations
While the catches of tsetse from monitoring traps declined following the deployment of targets, the age structure of the tsetse population did not change. This contrasts with findings in Zimbabwe where deployment of odour-baited targets had a marked effect on the age structure of a tsetse population [28]. Six months after the deployment of targets, the mean age class was 0.8-1.8 compared to 3.8 before targets were deployed. Analyses of the data from Zimbabwe suggested that mean daily mortality was between 4-10% where targets were present compared to 1-3% in their absence. Our estimates are based on dissections of 8308 tsetse compared to 1069 in the Zimbabwe-based study and hence it seems unlikely that the absence of any effect in the present study is due to a small sample size. A limitation in our study of age structure is that tsetse were not collected from the centre of the intervention area where we would expect the highest level of control and low levels of reinvasion. However, the low numbers of tsetse caught from these areas (e.g., 78 tsetse caught in the Kubala block in 2016) hampers robust estimates of age structure.

For all phases of the intervention, the catch of tsetse from monitoring traps generally declined to ≤1 tsetse/trap/day but did not reach and remain at zero in any parts of the intervention area. This can be explained most plausibly by invasion of tsetse from neighbouring areas where targets were not deployed. The fact that Tiny Targets caused a sharp decline in the density of tsetse populations but did not eliminate them completely, or change their age structure and infection status, confirmed the expectation that invasion can occur from either tsetse-infested areas outside or untreated drainages within the intervention area. The capture of large numbers of old flies (ovarian category >4) is consistent with invasion, since invading flies must be old.

Effective life of Tiny Targets
The persistence of low numbers of tsetse across the intervention area may have been assisted by the effective life of the targets being lower than predicted. The effective life of Tiny Targets was reported to be six months in a small-scale (<1 km2) field trial on an island in Lake Victoria [17]. In the present study, the survival of targets in the field was much less. In Maracha for instance, where catches persisted at about ~1 tsetse/trap/day, only 25% of targets inspected were functional (Fig. 6). Previous modelling of the impact of Tiny Targets [17] suggested that the mortality imposed by targets was ~4%/day and hence an 80% reduction in the density of functioning targets would reduce this to ~1%/day which is below the imposed mortality (4%/day) required to eliminate tsetse [37]. Nonetheless, imposing a mortality of 1%/day is predicted to reduce tsetse populations by about 90% but not lead to local elimination of tsetse [12]. However, the elimination of tsetse is not a pre-requisite for the elimination of gHAT. Mathematical models of gHAT [3, 4, 17, 38] and empirical studies [4, 19, 23] suggest that >70% reduction in abundance of tsetse will interrupt transmission.

Rivers in north west Uganda are prone to seasonal floods following heavy rain and these may be an important cause of the 27% of targets that went missing. We have also noticed that during the dry season, livestock visiting the river to drink also knock over targets. Some of these losses might be mitigated by placing the targets away from livestock watering points or on elevated places less vulnerable to flooding. Measures to prolong the effective life of targets must however be balanced by the need to ensure that they are in places where tsetse are present. The persistence of low numbers of tsetse across the Phase 3 intervention area may also be a result of the patchy distribution of targets. As is clear in Fig. 1F, the targets were deployed along only the larger rivers (>2 m wide) and it may be that small self-sustaining populations of tsetse persist within the intervention area.

Limitations
The present analysis aimed to use monitoring data from a national control programme to answer questions about the impact of scale-up on tsetse control. The scaling up and monitoring were not designed explicitly to address these research questions. Stronger inferences and conclusions could be produced by designing trials and sampling frameworks for this specific purpose [39]. For instance, the evidence that interventions in one tributary do not affect tsetse in an adjacent one could be strengthened by repeating the study across several paired rivers and designing the sampling framework so that each river was sampled simultaneously before and after targets were deployed, considering additional factors such as the distance between perennial rivers. Conclusions about the impact of target operational longevity on levels of control could be tested by comparing different densities of target with frequent monitoring of the targets to ensure that the effective densities are quantified. Monitoring of tsetse populations was carried out on the rivers where targets were deployed and we did not quantify the impact on tributaries and smaller streams. Tsetse populations on these smaller rivers are likely to be less affected by the deployment of Tiny Targets and hence the overall reduction in the tsetse population may be less than that inferred by catches from our monitoring traps.

Conclusions
Current practices for deploying Tiny Targets in Uganda reduces the density of tsetse by >80% which has contributed to a sustained reduction in the incidence of gHAT [23]. The impact on gHAT can be achieved without requiring local elimination of tsetse. Even better control of tsetse might be achieved by (i) deploying targets in places where they are less likely to be damaged by livestock and seasonal floods and (ii) deploying targets on more rivers, including small streams. However, the benefits of better control need to be balanced against the increased costs of deploying more targets. In the context of Uganda, where incidence of gHAT is exceedingly low, deploying targets on smaller rivers may not be cost-effective.

Acknowledgements
We thank the District Entomologists and Animal Production Officers, particularly Tom Onzivua and Stephen Onzima (Koboko), Harriet Batreru (Maracha), Philliam Cema (Arua) and Rashid Kawawa (Yumbe) and their teams who deployed Tiny Targets. We also thank the field technicians, Henry Ombanya, Mathia Amayo Erindia, Ignatius Jurua, Mark Ojoma and Patrick Edema who operated the network of monitoring traps, and the laboratory technicians, Victor Drapari, Edward Aziku and Alex Trima who performed the ovarian and infection dissections.

References
1. Franco JR, Cecchi G, Paone M, Diarra A, Grout L, Kadima Ebeja A, et al. The elimination of human African trypanosomiasis: Achievements in relation to WHO road map targets for 2020. PLOS Neglected Tropical Diseases. 2022;16(1):e0010047. doi: 10.1371/journal.pntd.0010047.

2. Rogers D, Robinson T. Tsetse Distribution. In: Maudlin I, Holmes P, Miles M, editors. The Trypanosomiases. Wallingford, UK: CABI Publishing; 2004. p. 139-79.

3. Rock KS, Torr SJ, Lumbala C, Keeling MJ. Predicting the Impact of Intervention Strategies for Sleeping Sickness in Two High-Endemicity Health Zones of the Democratic Republic of Congo. PLoS neglected tropical diseases. 2017;11(1). doi: 10.1371/journal.pntd.0005162. PubMed PMID: WOS:000394152000010.

4. Mahamat MH, Peka M, Rayaisse JB, Rock KS, Toko MA, Darnas J, et al. Adding tsetse control to medical activities contributes to decreasing transmission of sleeping sickness in the Mandoul focus (Chad). PLoS Negl Trop Dis. 2017;11(7):e0005792. Epub 2017/07/28. doi: 10.1371/journal.pntd.0005792. PubMed PMID: 28750007; PubMed Central PMCID: PMCPMC5549763.

5. Lord JS, Lea RS, Allan FK, Byamungu M, Hall DR, Lingley J, et al. Assessing the effect of insecticide-treated cattle on tsetse abundance and trypanosome transmission at the wildlife-livestock interface in Serengeti, Tanzania. PLoS Negl Trop Dis. 2020;14(8):e0008288. Epub 2020/08/26. doi: 10.1371/journal.pntd.0008288. PubMed PMID: 32841229; PubMed Central PMCID: PMCPMC7473525.

6. Hargrove JW, Ouifki R, Kajunguri D, Vale GA, Torr SJ. Modeling the control of trypanosomiasis using trypanocides or insecticide-treated livestock. PLoS Negl Trop Dis. 2012;6(5):e1615. Epub 2012/05/23. doi: 10.1371/journal.pntd.0001615. PubMed PMID: 22616017; PubMed Central PMCID: PMCPMC3352824.

7. Torr SJ, Hargrove JW, Vale GA. Towards a rational policy for dealing with tsetse. Trends Parasitol. 2005;21(11):537-41. Epub 2005/09/06. doi: 10.1016/j.pt.2005.08.021. PubMed PMID: 16140579.

8. Torr SJ, Vale GA. Is the even distribution of insecticide-treated cattle essential for tsetse control? Modelling the impact of baits in heterogeneous environments. PLoS Negl Trop Dis. 2011;5(10):e1360. Epub 2011/10/27. doi: 10.1371/journal.pntd.0001360. PubMed PMID: 22028944; PubMed Central PMCID: PMCPMC3196476.

9. Hargrove JW. A theoretical study of the invasion of cleared areas by tsetse flies (Diptera: Glossinidae). Bull Entomol Res. 2000;90(3):201-9. Epub 2000/09/21. doi: 10.1017/s0007485300000328. PubMed PMID: 10996861.

10. Vale GA, Hargrove JW, Solano P, Courtin F, Rayaisse JB, Lehane MJ, et al. Explaining the host-finding behavior of blood-sucking insects: computerized simulation of the effects of habitat geometry on tsetse fly movement. PLoS Negl Trop Dis. 2014;8(6):e2901. Epub 2014/06/13. doi: 10.1371/journal.pntd.0002901. PubMed PMID: 24921243; PubMed Central PMCID: PMCPMC4055578.

11. Vale GA, Hursey BS, Hargrove JW, Torr SJ, Allsopp R. The use of small plots to study populations of tsetse (Diptera: Glossinidae) - difficulties associated with populations dipsersal. Insect Sci Appl. 1984;5(5):403-10. PubMed PMID: ISI:A1984TZ39000014.

12. Vale GA, Torr SJ. Development of bait technology to control tsetse. In: Maudlin I, Holmes PH, Miles MA, editors. The Trypanosomiases Wallingford: CABI; 2004. p. 509-23.

13. Vale GA, Lovemore DF, Flint S, Cockbill GF. Odor-Baited Targets to Control Tsetse Flies, Glossina Spp (Diptera, Glossinidae), in Zimbabwe. Bulletin of Entomological Research. 1988;78(1):31-49. doi: Doi 10.1017/S0007485300016059. PubMed PMID: WOS:A1988M431000004.

14. Rayaisse JB, Tirados I, Kaba D, Dewhirst SY, Logan JG, Diarrassouba A, et al. Prospects for the development of odour baits to control the tsetse flies Glossina tachinoides and G. palpalis s.l. PLoS Negl Trop Dis. 2010;4(3):e632. Epub 2010/03/20. doi: 10.1371/journal.pntd.0000632. PubMed PMID: 20300513; PubMed Central PMCID: PMCPMC2838779.

15. Omolo MO, Hassanali A, Mpiana S, Esterhuizen J, Lindh J, Lehane MJ, et al. Prospects for developing odour baits to control Glossina fuscipes spp., the major vector of human African trypanosomiasis. PLoS Negl Trop Dis. 2009;3(5):e435. Epub 2009/05/13. doi: 10.1371/journal.pntd.0000435. PubMed PMID: 19434232; PubMed Central PMCID: PMCPMC2674566.

16. Rogers D. Study of a Natural-Population of Glossina-Fuscipes-Fuscipes Newstead and a Model of Fly Movement. J Anim Ecol. 1977;46(1):309-30. doi: Doi 10.2307/3962. PubMed PMID: WOS:A1977CU67200020.

17. Tirados I, Esterhuizen J, Kovacic V, Mangwiro TN, Vale GA, Hastings I, et al. Tsetse Control and Gambian Sleeping Sickness; Implications for Control Strategy. PLoS Negl Trop Dis. 2015;9(8):e0003822. Epub 2015/08/13. doi: 10.1371/journal.pntd.0003822. PubMed PMID: 26267814; PubMed Central PMCID: PMCPMC4580652.

18. Lindh JM, Torr SJ, Vale GA, Lehane MJ. Improving the cost-effectiveness of artificial visual baits for controlling the tsetse fly Glossina fuscipes fuscipes. PLoS Negl Trop Dis. 2009;3(7):e474. Epub 2009/07/08. doi: 10.1371/journal.pntd.0000474. PubMed PMID: 19582138; PubMed Central PMCID: PMCPMC2699553.

19. Courtin F, Camara M, Rayaisse JB, Kagbadouno M, Dama E, Camara O, et al. Reducing Human-Tsetse Contact Significantly Enhances the Efficacy of Sleeping Sickness Active Screening Campaigns: A Promising Result in the Context of Elimination. PLoS Negl Trop Dis. 2015;9(8):e0003727. Epub 2015/08/13. doi: 10.1371/journal.pntd.0003727. PubMed PMID: 26267667; PubMed Central PMCID: PMCPMC4534387.

20. Tirados I, Hope A, Selby R, Mpembele F, Miaka EM, Boelaert M, et al. Impact of tiny targets on Glossina fuscipes quanzensis, the primary vector of human African trypanosomiasis in the Democratic Republic of the Congo. PLoS Negl Trop Dis. 2020;14(10):e0008270. Epub 2020/10/17. doi: 10.1371/journal.pntd.0008270. PubMed PMID: 33064783; PubMed Central PMCID: PMCPMC7608941 Boelaert and Erick Mwamba Miaka were unable to confirm their authorship contributions. On their behalf, the corresponding author has reported their contributions to the best of their knowledge.

21. Kaba D, Djohan V, Berte D, Ta BTD, Selby R, Kouadio KAM, et al. Use of vector control to protect people from sleeping sickness in the focus of Bonon (Cote d'Ivoire). PLoS Negl Trop Dis. 2021;15(6):e0009404. Epub 2021/06/29. doi: 10.1371/journal.pntd.0009404. PubMed PMID: 34181651; PubMed Central PMCID: PMCPMC8238187 Rayaisse was unable to confirm their authorship contributions. On their behalf, the corresponding author has reported their contributions to the best of their knowledge.

22. Courtin F, Dupont S, Zeze DG, Jamonneau V, Sane B, Coulibaly B, et al. [Human African trypanosomiasis: urban transmission in the focus of Bonon (Cote d'Ivoire)]. Trop Med Int Health. 2005;10(4):340-6. Epub 2005/04/06. doi: 10.1111/j.1365-3156.2005.01398.x. PubMed PMID: 15807798.

23. Bessell P, Esterhuizen J, Lehane M, Longbottom J, Mugenyi A, Selby R, et al. Estimating the impact of Tiny Targets in reducing the incidence of gambiense sleeping sickness in the North West Uganda focus. Parasites & Vectors. 2021;14:999-1000.

24. Ndung'u JM, Boulange A, Picado A, Mugenyi A, Mortensen A, Hope A, et al. Trypa-NO! contributes to the elimination of gambiense human African trypanosomiasis by combining tsetse control with "screen, diagnose and treat" using innovative tools and strategies. PLoS Negl Trop Dis. 2020;14(11):e0008738. Epub 2020/11/13. doi: 10.1371/journal.pntd.0008738. PubMed PMID: 33180776; PubMed Central PMCID:

PMCPMC7660505 manufactures the Tiny Targets used in this project. They however play no role in implementation of the project.

25. Shaw AP, Tirados I, Mangwiro CT, Esterhuizen J, Lehane MJ, Torr SJ, et al. Costs of using "tiny targets" to control Glossina fuscipes fuscipes, a vector of gambiense sleeping sickness in Arua District of Uganda. PLoS Negl Trop Dis. 2015;9(3):e0003624. Epub 2015/03/27. doi: 10.1371/journal.pntd.0003624. PubMed PMID: 25811956; PubMed Central PMCID: PMCPMC4374750.

26. Kovacic V, Tirados I, Esterhuizen J, Mangwiro CT, Torr SJ, Lehane MJ, et al. Community acceptance of tsetse control baits: a qualitative study in Arua District, North West Uganda. PLoS Negl Trop Dis. 2013;7(12):e2579. Epub 2013/12/19. doi: 10.1371/journal.pntd.0002579. PubMed PMID: 24349593; PubMed Central PMCID: PMCPMC3861179.

27. Vale GA, Torr SJ. User-friendly models of the costs and efficacy of tsetse control: application to sterilizing and insecticidal techniques. Med Vet Entomol. 2005;19(3):293-305. Epub 2005/09/02. doi: 10.1111/j.1365-2915.2005.00573.x. PubMed PMID: 16134978.

28. Van Sickle J, Phelps RJ. Age distributions and reproductive status of declining and stationary populations of Glossina pallidipes Austen (Diptera: Glossinidae) in Zimbabwe. Bulletin of Entomological Research. 2009;78(1):51-61. doi: 10.1017/s0007485300016060. PubMed PMID: WOS:A1988M431000005.

29. WHO. Global Health Observatory data repository - Human African Trypanosomiasis [Internet] 2020 [cited 2022 3 Feb 2022]. Available from: http://apps.who.int/gho/data/node.main.A1635?lang=en.

30. Gilbert M, Nicolas G, Cinardi G, Van Boeckel TP, Vanwambeke SO, Wint GRW, et al. Global distribution data for cattle, buffaloes, horses, sheep, goats, pigs, chickens and ducks in 2010. Sci Data. 2018;5(180227):180227. Epub 2018/10/31. doi: 10.1038/sdata.2018.227. PubMed PMID: 30375994; PubMed Central PMCID: PMCPMC6207061.

31. Clausen PH, Adeyemi I, Bauer B, Breloeer M, Salchow F, Staak C. Host preferences of tsetse (Diptera: Glossinidae) based on bloodmeal identifications. Med Vet Entomol. 1998;12(2):169-80. Epub 1998/06/11. doi: 10.1046/j.1365-2915.1998.00097.x. PubMed PMID: 9622371.

32. Lindh JM, Goswami P, Blackburn RS, Arnold SE, Vale GA, Lehane MJ, et al. Optimizing the colour and fabric of targets for the control of the tsetse fly Glossina fuscipes fuscipes. PLoS Negl Trop Dis. 2012;6(5):e1661. Epub 2012/06/06. doi: 10.1371/journal.pntd.0001661. PubMed PMID: 22666511; PubMed Central PMCID: PMCPMC3362611.

33. Gouteux JP, Lancien J. The pyramidal trap for sampling and controlling tsetse flies (Diptera: Glossindae) comparative trials and descriptions of new collecting techniques. . Tropical Medicine and Parasitology. 1986;37(1):61-6. PubMed PMID: WOS:A1986A785400013.

34. R TC. R: A language and environment for statistical computing. R Foundation for Statistical Computing. Vienna, Austria2020.

35. Stanton MC, Esterhuizen J, Tirados I, Betts H, Torr SJ. The development of high resolution maps of tsetse abundance to guide interventions against human African

trypanosomiasis in northern Uganda. Parasit Vectors. 2018;11(1):340. Epub 2018/06/10. doi: 10.1186/s13071-018-2922-5. PubMed PMID: 29884213; PubMed Central PMCID: PMCPMC5994020.

36. Vreysen MJ, Balenghien T, Saleh KM, Maiga S, Koudougou Z, Cecchi G, et al. Release-recapture studies confirm dispersal of Glossina palpalis gambiensis between river basins in Mali. PLoS Negl Trop Dis. 2013;7(4):e2022. Epub 2013/05/02. doi: 10.1371/journal.pntd.0002022. PubMed PMID: 23634232; PubMed Central PMCID: PMCPMC3636245.

37. Hargrove JW. Tsetse: the limits to population growth. Med Vet Entomol. 1988;2(3):203-17. Epub 1988/07/01. doi: 10.1111/j.1365-2915.1988.tb00184.x. PubMed PMID: 2980177.

38. Pandey A, Atkins KE, Bucheton B, Camara M, Aksoy S, Galvani AP, et al. Evaluating long-term effectiveness of sleeping sickness control measures in Guinea. Parasit Vectors. 2015;8:550. Epub 2015/10/23. doi: 10.1186/s13071-015-1121-x. PubMed PMID: 26490037; PubMed Central PMCID: PMCPMC4618537.

39. Longbottom J, Krause A, Torr SJ, Stanton MC. Quantifying geographic accessibility to improve efficiency of entomological monitoring. PLoS Negl Trop Dis. 2020;14(3):e0008096. Epub 2020/03/24. doi: 10.1371/journal.pntd.0008096. PubMed PMID: 32203517; PubMed Central PMCID: PMCPMC7117774.

Click here to access/download
Supporting Information
SI - original data.xlsx
