# Supplementary material for: Scaling up of tsetse control to eliminate Gambian sleeping sickness in northern Uganda
Source: PLoS Negl Trop Dis. 2022 Jun 29;16(6):e0010222. doi: 10.1371/journal.pntd.0010222 (PMC9275725; doi:10.1371/journal.pntd.0010222)
Supplement: Attachment — Submitted filename: Response to Reviewers comments.docx [file pntd.0010222.s003.docx]

|  | Comment | Response |
| --- | --- | --- |
| R1 | 1. The authors need to think more carefully about why it is possible to get away with "sparse deployment of baits". To my mind this is not simply because of the dispersal propensity of tsetse. It is also a function of the extremely low rate of reproduction in tsetse - which means that we only need to kill 3.5% of adult females per day in order to send the population into negative growth. Rewrite the appropriate text, referring to the appropriate papers. | See response to ‘pdf’ above |
|  |  |  |
| R1 | 2. The authors seem to get confused between “square km” [km2] and “per square km” [km-2]. Please check throughout. | We have reviewed all cases of km2 and believe these to be correct. |
|  |  |  |
| R1 | 3. Please do a proper statistical analysis of the data in Table 1, providing estimates, 95% confidence intervals and appropriate statistical analyses, and an interpretation of is happening. | See response to Reviewer 3 below. |
|  |  |  |
| R1 | 4. On the statistics front, it is also insufficient to provide an F statistic and a P value: the relation between the two is a function of the degrees of freedom, which must therefore be stated. | We have added degrees of freedom |
|  |  |  |
| R1 | Also attached is a copy of the pdf of the paper – with edits attached that the authors should address. | See responses to ‘pdf’ below |
| pdf | Italics? | Correct in main MS. We believe this is an artefact of the online submission process where the abstract is pasted into requisite boxes. We will check when as we resubmit. |
|  | Does this mean "per sq km"? | No, it means 250 square kilometres. We think this and other queries about formatting on the cover sheet above are caused by the system. We believe that the formatting is correct in the submitted MS. |
| pdf | sub-sample | Corrected to ‘sub-samples’ (P2, line 16) |
| pdf | should or would? | Changed to ‘might’ (P2, line 29) |
| pdf | This says "1600 square km". I presume you want to say "1600 PER sq km" | The intention is indeed to say 1600 square kilometres and we have therefore not changed the text.  We have also checked all other questions about km2 and believe these to be correct. |
| pdf | Rather more complicated than this, isn't it. We can only get away with "sparse deployment of baits" because of the extremely low rate of reproduction in tsetse - which means that we only need to kill 3.5% of adult females per day in order to send the population into negative growth. Rewrite this sentence, referring to the appropriate papers. | We have re-written this paragraph, focussing on riverine tsetse and highlighting:- the limits to growth and the relevant paper by Hargrove, the mobility of riverine tsetse (papers by Rogers and Hargrove) allowing a sparse deployment of targets, concentration of G. fuscipes tsetse along rivers, and reduction rather than elimination of tsetse can lead to interruption of transmission. (P4, lines 28-47) |
| pdf | Ugly | Removed but the argument is retained in the re-written paragraph. |
| pdf | Please go through the whole paper and sort out this repeated problem. | We have done so and believe all to be correct. |
| pdf | ~~Re-~~invasion | Changed to ‘re-invasion’ to ‘invasion’ throughout |
| pdf | impact ~~on~~ | We believe ‘impact on’ rather than ‘impact’ is correct in this instance. |
| pdf | Not clear where the Figure legend ends. Bits at the top of the next page look as if they SHOULD be part of the legend - but not clear .... | We have moved all figures to single pages at the end of the manuscript so that it is clear that the text is part of the caption. |
| pdf | Perhaps: "Inset shows, schematically, locations " etc ..... | The inset has been removed from Fig.2 and is now Figure 1 in the SI document. We have written ‘schematically’ as suggested. |
| pdf | Do the analyses and I think you will find that there ARE some significant differences.  [See the example for the midgut data - assuming I have interpreted properly what the numbers mean ....]  Either way, do the analyses properly. | See response to Reviewer 3 below. As suggested by both reviewers we have re-done a fuller and correct statistical analysis of the data following the example shown. |
| pdf | Degrees of freedom please | Degrees of freedom added (P23, lines 10, 13 and 16). |
| pdf | Clarify whether, in fact, you are meaning impact or LACK of impact.....  For the Rifa Triangle study (Vale et al) it was surprising that reductions in trap catch could only be seen in traps for <5km into the main invasion front.  BUT, according to Zambian reports, use of targets in the Rifa caused the collapse of the tsetse populations on the other side of the Zambezi. | Text changed to make it clearer that other studies have found impact beyond where targets were deployed.  “Previous studies with riverine [22] and savanna [40] tsetse have indicated that targets reduce the abundance of the flies outside the intervention area.” (P 14, lines 6-8) |
| pdf | Too strong I think. Perhaps something like "invading flies will naturally tend to be older on average" | Re-written as follows:-  “The capture of large numbers of old flies (ovarian category >4) is consistent with invasion, since it takes time for tsetse to invade.” (P15, lines 31-32) |
|  |  |  |
| R3 | These are likely completed correctly, but the writing needs improvement in organization to make this adequate for publication. See details below. | See responses below. |
|  |  |  |
| R3 | The conclusion paragraph is not appropriate, it is not limited to findings from this study and does not address the major questions set out to answer. | The concluding paragraph has been changed so that they relate directly to the four research questions listed in the Introduction. (P16, lines 28-46) |
|  |  |  |
| R3 | 1) The intro does not introduce all concepts necessary to understand the importance of some components of the study. Several of the following points are examples of this. | See responses to each component listed by the reviewer below. |
|  |  |  |
| R3 | The importance and implications of up vs downstream measurements is unclear, more explanation is needed in the intro and discussion to make it clear why you measure this, and what the results mean. | We have added a section in the Introduction (P 5, lines 27-36) and in the relevant section of the Materials and Methods (P9, lines 13-22). |
|  |  |  |
| R3 | 2) Especially in the intro, statements about 70% suppression being sufficient to break the transmission cycle are not well explained or supported. Even after reading the discussion where 6 papers are cited, I am not convinced that this threshold is broadly well-supported. This is a complicated issue, with some not-so-intuitive responses in infection rate. For instance, isn’t there a reduction in dilution effect with fewer flies, which would lead to short-term increased infection rates very locally around the infected hosts? Furthermore, results of infection rates do not support this assertion, yet it comes up again in the conclusion repeating the statement as if it were a fact. | In the Introduction we have added to the single paper originally cited and added references to theoretical and empirical evidence that a reduction in the abundance of tsetse can lead to reductions in incidence. (P5, lines 12-25)  Regarding impacts on dilution effect, this is an interesting suggestion that merits empirical testing. For the particular case of gHAT in northern Uganda, with highly mobile vectors, no evidence that there are important animal reservoir hosts, we do not think that there will be very local effects around infected hosts (ie, humans). We only found one tsetse with a mature *T. brucei s.l.* infection and we do not think that our data allows us to test this hypothesis.  For a fuller analysis of the infection rates and our interpretation of these please see the response below |
|  |  |  |
| R3 | I think this issue needs to be much more carefully stated from the beginning, with the source of the statement clearly laid out with caveats acknowledged (even in the abstract). |  |
|  |  |  |
| R3 | Then it should be removed from the conclusion, and you should replace this with conclusions drawn from the present study. | Please see revised conclusions |
|  |  |  |
| R3 | 3) My sense is that this 70% suppression threshold is from mathematical models with some empirical evidence in some settings, but real-world stochasticity and spatial heterogeneity alters this threshold and the dynamics of this variation have not yet been adequately studied. If this is true, then the logic should be put to use to explain the importance of testing this threshold, and your data can inform this. | In the Introduction, we have provided a fuller consideration of theoretical (modelling) and empirical papers related to the suppression threshold. Rather than arguing that there is a universal and unique threshold where transmission of gHAT is interrupted, we focus on the evidence that transmission can be interrupted without eliminating a tsetse population. Our study contributes empirical data to quantify the impact of tsetse control in northern Uganda. (P5, lines 12-25) |
|  |  |  |
| R3 | In my opinion, predictions of infection rates should be more clearly motivated in the intro, and testing the prediction of reduced infection rates should be included in the stated goals (otherwise, why do you go through the trouble of estimating them?). | We have added a section outlining how vector control might impact on the age structure in infection rates of tsetse populations. (P5, lines 38-45) |
|  |  |  |
| R3 | 4) On this topic, I suspect that there is a lot of interesting detail in your results that was lost when you averaged across the full study area for Table 1. Can you separate by drainage? | We have reanalysed the data to take account of drainages. (P12, lines 15-43) |
|  |  |  |
| R3 | Can you even compare infection rates in different areas of suppression (inside+edge vs outside for example?). This would be really interesting! | We have re-done the analyses as suggested. (P12, lines 33-43) |
|  |  |  |
| R3 | And why did you split everything up by body parts? There is probably really interesting reasons for doing this, so can you explain and include these details? | We have explained the methods and their rationale in the Materials and Methods. (P8, lines 30-42) |
|  |  |  |
| R3 | Does separating by drainage help explain the mismatch of infection status in midgut vs mouthparts? Or are their other caveats such as miss-scoring of any of these body parts that might explain the mismatch? | We have redone the analyses, taking account of drainages, as suggested. The results do not explain the mismatch but we do discuss this in the Results and Discussion |
|  |  |  |
| R3 | I would find these results and discussions very interesting. I know that you are dealing with small sample sizes, but with the data I can see (original data does not specify which drainage or which treatment area the flies are from for Table 1), it seems like there could be more meaningful interpretation can be made. | We have re-done the analyses and presented the results. (P12, lines 33-43) |
|  |  |  |
| R3 | 5) In the intro, there is no clear set-up of why age structure is expected to change (this is buried in the discussion and should be moved to the intro). Further, the prediction is lumped in with the stated goal of how the expansion of tsetse control operations impacted levels of suppression. You would be better off dedicating a paragraph in the intro and a stand-alone research question. | In the Introduction, we have added a paragraph providing theoretical and empirical evidence for expecting tsetse control to affect age structure. (P5, lines 38-45)  We have also re-analysed the ovarian category data according to drainage so that it matches the analyses of infection data. (P7, lines 15-31)  We have increased the number of research questions and re-phrased the question that considers impact on age structure and infection rates (P6, lines 15-24) |
|  |  |  |
| R3 | 6) The discussion does not clearly answer the questions laid out in the intro and methods. This problem is especially evident in the conclusion, which does not address the central issue presented in the summary, abstract and intro (expansion of the intervention area). I would encourage the authors to build a coherent outline that is then addressed in each section: intro, methods, results, discussion. Usually this works best if you keep the order logical and consistent (although exceptions can flow better, depending on the details, especially in the discussion). | We have made a clearer link between the four research questions first introduced in the Introduction which we then follow in the Methods, Results, Discussion and Conclusion. |
|  |  |  |
| R3 | 7) The methods are not complete. Where is the description of how a “functional trap” is defined and measured?  Where is the info about how age was determined, and details on how infection status was determined in each body part? Most importantly, the different statistical tests performed to answer each question are not clear. I would strongly advise stating the methods and predictions for each question/hypothesis, including the two that are missing sub-headings (age structure and infection status). Then the section describing the statistical methods can be a short section describing what was done when not otherwise noted and graphical methods, or it can be deleted. Finally, when reporting the “significance”, the statistical test performed, relevant settings and sampling design, and relevant results (such as p-values, etc.) should always be reported along with the figure and table with the data that was tested. Even in the discussion, the data used and result referenced should be apparent when you discuss each result. Add references to the appropriate figures and tables throughout. | In the materials and methods we have:-  Defined a ‘functional target’ (“Targets that were present, upright and undamaged were regarded as functional.”) (P9, lines9-10)  Added details of how age and infection status were determined. (P8, lines 30-42)  Added a section entitled ‘Experimental design’ which comprises four paragraphs describing the design and predictions for each research question. (P9, line 12 - p10, line 2)  Provided details of statistical methods for each research question. We considered carefully the suggestion of moving the statistical methods to each research question but felt that this would lead to repetition for questions 1-3. We have therefore retained the section on statistical methods but added more details of the statistical methods. We hope the changes address the reviewer’s concerns. (P10, lines 4-33)  In the Results, we have:-  Added three new Tables in the SI with details of the means, errors, statistical tests related to catches of tsetse. We have also added a figure in the main text that shows more clearly the reductions achieved.  The text has been adjusted to take account of the new tables and figures. |
|  |  |  |
| R3 | 8) There is a big issue here in study design because there is no control for any of the tests. Each of the comparisons made is confounded by differences in time or space. If I am understanding your experimental set-up correctly, there are no “centers” of treatment that did not get expanded treatment, so there is no control for the main question of the study. Thus, the centers have differences in time since beginning treatment as well as being in the center There is a similar problem (this time confounded by time) in comparison of the drainage with/without treatment, the no-treatment drainage was not sampled pre-treatment along with the other drainages, so it cannot serve as a true control. I see that you put a section that discusses limitations, and although I think this can be a part of your strategy to deal with these issues, I do not think it is sufficient on its own. I think you need to add details of the limitations for each research question right up-front where you discuss the experimental design and the statistical tests used. This way the reader can understand what is the appropriate interpretation of each of your results. | We agree that the study design was unclear and that we did not adequately address the confounding of time and space. The Methods have been adjusted to highlight this difficulty and how we approached this. (See “Experimental design”; P9, line 12 – P10, line 2)  We have added a graph to Fig. 2 showing the results from Koboko, where no targets, were deployed for Phases 1 and 2. To quantify the impact of Tiny Targets we compare longitudinal changes in catches of tsetse from areas with and without targets.  The results for statistical tests for different locations and Districts with and without targets are presented in new Tables (SI, Tables 1-3). The results show a significant decline in places where targets were present but not where they were absent. |
|  |  |  |
|  | Minor comments: |  |
| R3 | 9) Mapping methods for rivers. What are you sources and methods? For example, how do you make the major rivers thicker lines on the maps than the minor tributaries? In my understanding, this is not simple, can you give all details to make it reproducible for future researchers? | In the Methods, we have added a section describing the source of the river shapefile and how we varied the thickness of the lines according to stream level. (P8, lines 48-50) |
|  |  |  |
| R3 | 10) Line numbers missing. I would have some grammatical and editorial comments if there were line numbers, next time make sure you add those to your document! I am unable to spend the time explaining the location of each of my edits without them at this time. | Sorry! We have added line numbers to the re-submitted manuscript. |
|  |  |  |
| R3 | 11) It would be good to see a report of the deployment data, this would allow for discussion of “incomplete deployment of targets in mid-2014”. As written, this comes out of nowhere and is not supported by data. | We have added a report in the SI on the deployment of targets in 2014. |
|  |  |  |
| R3 | 12) Are 1 and 0.1 catches per day biologically relevant? If so explain, if not, then I would encourage you to use more descriptive language that describes the actual levels rather than saying above/below these arbitrary thresholds. Additionally, are the “general patterns” described statistically significant differences? It is not clear from looking at the figure how significantly different they may or may not be, and then the description using the thresholds makes it possible these are arbitrary differences that are not statistically or biologically significant. | We have changed the text to report actual levels and the new tables show which differences are statistically significant.  A new figure (Fig. 3) provides a clearer indication of the general trends implicit in Fig. 2. |
|  |  |  |
| R3 | 13) I would like to see a paragraph describing general trapping results before addressing the tests to your hypotheses. This would improve the flow and the readability. | We have reorganised the Introduction, Methods and Results so that they show a clearer link to the research questions. We hope that this improves the overall flow. |
|  |  |  |
| R3 | 14) There are several statements in the results that should be put in the discussion, and are also do not logically flow.  a. “The higher catches on the Oluffe suggests…” Given limitations, this statement is basically speculation and belongs in the discussion with caveats. Further, it is stated before the relevant results are presented.  b. “Overall, the results suggest that the effects of targets…” Belongs in the discussion. | For a. and b., we have moved and re-written the statements to relevant parts of the Discussion |
